# Association of the Vaginal Microbiota with Human Papillomavirus Infection in a Korean Twin Cohort

**DOI:** 10.1371/journal.pone.0063514

**Published:** 2013-05-22

**Authors:** Jung Eun Lee, Sunghee Lee, Heetae Lee, Yun-Mi Song, Kayoung Lee, Min Ji Han, Joohon Sung, GwangPyo Ko

**Affiliations:** 1 Department of Environmental Health and Institute of Health and Environment, School of Public Health, Seoul National University, Seoul, Republic of Korea; 2 Department of Family Medicine, Samsung Medical Center, Sungkyunkwan University School of Medicine, Seoul, Republic of Korea; 3 Department of Family Medicine, Busan Paik Hospital, Inje University College of Medicine, Busan, Republic of Korea; 4 Department of Epidemiology, School of Public Health, Seoul National University, Seoul, Republic of Korea; IPO, Inst Port Oncology, Portugal

## Abstract

Human papillomavirus (HPV) is the most important causative agent of cervical cancers worldwide. However, our understanding of how the vaginal microbiota might be associated with HPV infection is limited. In addition, the influence of human genetic and physiological factors on the vaginal microbiota is unclear. Studies on twins and their families provide the ideal settings to investigate the complicated nature of human microbiota. This study investigated the vaginal microbiota of 68 HPV-infected or uninfected female twins and their families using 454-pyrosequencing analysis targeting the variable region (V2–V3) of the bacterial 16S rRNA gene. Analysis of the vaginal microbiota from both premenopausal women and HPV-discordant twins indicated that HPV-positive women had significantly higher microbial diversity with a lower proportion of *Lactobacillus* spp. than HPV-negative women. Fusobacteria, including *Sneathia* spp., were identified as a possible microbiological marker associated with HPV infection. The vaginal microbiotas of twin pairs were significantly more similar to each other than to those from unrelated individuals. In addition, there were marked significant differences from those of their mother, possibly due to differences in menopausal status. Postmenopausal women had a lower proportion of *Lactobacillus* spp. and a significantly higher microbiota diversity. This study indicated that HPV infection was associated with the composition of the vaginal microbiota, which is influenced by multiple host factors such as genetics and menopause. The potential biological markers identified in this study could provide insight into HPV pathogenesis and may represent biological targets for diagnostics.

## Introduction

Vaginal microbiota play a crucial protective role in women’s health, and the host biochemical and immunological response to the microbiota can serve as an indicator of the health indicators of the vaginal environment [1]. A healthy microbiota prevents or hinders many urogenital diseases, such as yeast infections, sexually transmitted infections, urinary tract infections, and human immunodeficiency virus (HIV) infection [2], [3], [4], [5], [6]. An abnormal microbiota, such as that found in bacterial vaginosis (BV), is associated with an increased risk of upper genital tract health issues, miscarriage [7], recurrent abortion [8], preterm delivery [8], HPV infection [9], and sexually transmitted infections, including HIV [10]. A healthy vaginal microbiota is composed primarily of lactic acid-producing bacteria such as *Lactobacillus* spp., which are commonly found on the vaginal epithelium, and contribute to women’s health by maintaining a low pH in the vagina through lactic acid production. In addition, the microbiota inhibit competing bacterial taxa by producing bacteriocins, which kill closely related bacterial species, and biosurfactants, which prevent the attachment of other species to the epithelium [11]. With the advancement of sequencing technologies, tools for investigating the human microbiome in various body sites have become available [11]. Most previous research on the vaginal microbiota focused on the composition of the vaginal microbiota of healthy women or those with BV [1], [12], [13], although more recently, an association between the vaginal microbiota and HIV infected women was reported [14], [15], [16].

Cervical cancer is the third most common cancer in women worldwide [17], and human papillomaviruses (HPVs) are considered to be the most important sexually transmitted causal agent of cervical intraepithelial neoplasia (CIN) and cervical adenocarcinoma [18], [19], although the association with cervical cancer is confined to specific high-risk types of HPV. High-risk types of HPV are strongly associated with cervical cancer and are detected in 99% of cervical cancers, whereas low-risk types are associated with benign or less malignant genital warts [20]. HPVs are common in the anogenital mucosa of sexually active adults and adolescents, and most HPVs have been found in this body region [20], [21], [22]. The age-adjusted prevalence of HPV in women is estimated to be 10.4%, ranging from 8.1% in Europe to 22.1% in Africa [23]. To-date, age, sexual activity, menopausal status, and hormone therapy have been found to be associated with the development and persistence of HPV infection [23]. However, other factors associated with anogenital HPV infection are poorly understood, despite the vaginal microbiota, mucosal immunity, the level of estrogen hormones, and host genetic factors having been assumed to play important roles in HPV susceptibility.

Studies on twins and their mothers can provide valuable information for discriminating the effects of genetic and various environmental factors on vaginal microbiota [24], [25]. In this study, we investigated the association between the vaginal microbiota and HPV infection using a twin cohort with consideration of menopausal status. In addition, potential microbiological markers associated with HPV infection were investigated.

## Materials and Methods

### Study Population

This study was performed using cervicovaginal samples collected from 912 women who participated in the Healthy Twin Study, a part of the Korean Genome Epidemiology Study [26]. The zygosity of twins was tested using the Amp*Fl*STR Identifier Kit (Perkin Elmer, Waltham, MA, USA) with 16 short tandem repeat (STR) markers (15 autosomal STR markers and 1 sex-determining marker) in 67% of the twins. For the remaining 33%, a zygosity-determining questionnaire with a validated accuracy of >90% was applied [27]. All subjects were between 31 and 73 years of age. Written informed consent was obtained from each participant. The study protocol was approved by the Korea Centers for Disease Control and the institutional review board (IRB) of the three participating institutions: Samsung Medical Center, Inje University Busan Paik Hospital, and Seoul National University (IRB No. 144-2011-07-11).

### Sample Collection and HPV Screening

In the selection of these samples, we examined 912 women who had undergone a Pap smear test between 2005 and 2009 in South Korea as part of the health examination for the Healthy Twin Study. The average age of twins and siblings was 43 and that of their mothers was 65. Each cervicovaginal sample was collected with a separate endocervical brush. The liquid-based preparation method for collecting cervicovaginal samples was performed according to the manufacturer’s instructions (ThinPrep® and Surepath™) and stored until further analysis [28]. Whole genomic DNA was extracted from each of the 980 sample swabs collected from 912 twins, siblings, and their mothers; to determine the presence of HPV infection, 912 subjects were tested for HPV using two primer sets, MY09/MY11 and GP5+/GP6+, as described in a previous study [18]. PCR amplicons of 450 and 150 bp were sequenced for confirmation and subsequent typing of HPV (high *vs*. low risk). HPV genotypes 16, 18, 33, 35, 39, 45, 51, 52, 56, 58, 59, and 66 were identified as high risk and the others were identified as low risk. Among the 912 subjects, 72 (7.9%) were positive for HPV infection; of these 72 subjects, those who had received antibiotics in the 6-month period prior to specimen sampling were excluded.

In addition, patients with *Trichomonas vaginalis* infection and/or atypical cells of undetermined significance (ASCUS), as determined by clinical examination, were also excluded to eliminate possible confounding effects associated with these agents. As a result, we chose 23 HPV-positive and 45 HPV-negative subjects. The 23 HPV-positive subjects included 10 HPV-discordant MZ twins (HPV positive, without CIN [N = 9]; HPV positive with CIN [N = 1]), four HPV-positive concordant MZ twin pairs (both MZ twin pairs were HPV positive), five HPV-positive unpaired MZ or DZ twins (HPV-positive twins where the matching twin was excluded based on the criteria described above, N = 5), and four siblings (N = 4). The 45 HPV-negative subjects included HPV-discordant MZ twins (N = 10), HPV-negative concordant MZ twins (N = 2), DZ paired twin pair (N = 2), HPV-negative unpaired MZ twin (N = 6), siblings (N = 15), and mothers (N = 10). The epidemiological and clinical information of the study population are summarized in Table S1.

Among the study subjects (N = 68), 50 and 18 were pre- and postmenopausal women as identified by self reporting, respectively. Postmenopausal women included some of the twins (N = 4), their siblings (N = 4), and all of the twins’ mothers (N = 10). Among the 18 postmenopausal women, three had a history of hormone replacement therapy (HRT). To determine the effect of HPV infection on vaginal microbiota and women’s health, 50 subjects from three subgroups–HPV negative and CIN negative (N = 26), HPV positive and CIN negative (N = 19), and CIN positive (N = 5; four HPV-positive and one HPV-negative subjects)–were selected. Postmenopausal women and those with noticeable infection on physical examination were excluded from this portion of the study. We included only 26 women as HPV-negative healthy controls from the 50 premenopausal women after exclusion of subjects with HPV, other noticeable infections, or CIN. Among the five women with CIN, four were identified as HPV positive. For the analysis of HPV-discordant twins, only nine MZ twin pairs (N = 18) without CIN, who were 31−49 years old, were selected. One discordant twin pair with CIN was excluded from this analysis due to possible interference with clinical symptoms. Table 1 summarizes the study population.

**Table 1:** Summary of the study population (N = 68).

	Premenopausal women (N = 50)		Postmenopausal women (N = 18)		Sum
	HPV (−) (N = 27)	HPV (+) (N = 23)	HPV (−) (N = 18)	HPV (+) (N = 0)	
HPV-discordant MZ twinHPV-concordant MZ twinUnpaired MZ twinDZ twinSiblingMother	102(1)†31110	10(1)†43(1)†24(2)†0	0031410	000000	206941910
Sum	27	23	18	0	68

† The number with cervical intraepithelial neoplasia (CIN).

### DNA Extraction, PCR Amplification of 16S rRNA, and Purification

Total genomic DNA was extracted from each swab (N = 68) using an extraction kit. Genomic DNA was extracted from the cytobrush using the chemagic viral DNA/RNA kit (Chemagen, Baesweiler, Germany) according to the manufacturer’s instructions. The extraction method for total nucleic acids was compared with the conventional bead beating method using 16S rRNA amplification and DGGE analysis. No significant difference was observed. The extracted nucleic acids were stored at −70°C until use. The V2 and V3 regions of the 16S rRNA genes were integrally amplified by polymerase chain reaction (PCR) using the barcoded universal primers presented in Table S2, and pyrosequenced using the 454 Life Sciences FLX Titanium machine (Roche, Indianapolis, IN, USA). For each sample, we amplified the 16S rRNA genes using the primer set 8 forward and 534 reverse [29], [30]. The forward primer contained the 454 Life Sciences (Roche) Titanium-compatible adaptor sequences (5′-CCTATCCCCTGTGTGCCTTGGCAGTC-3′), a four-base linker sequence (“TCAG”), and the broadly conserved bacterial primer 8F (5′-AGAGTTTGATCCTGGCTCAG-3′). The reverse primer contained the 454 Life Sciences (Roche) Titanium-compatible adaptor sequences (5′-CCATCTCATCCCTGCGTGTCTCCGAC-3′), a four-base linker sequence (“TCAG”), and a 9- or 10-base multiplex identifier (MID) used to tag each PCR product, the bacterial primer 534R (5′-ATTACCGCGGCTGCTGG-3′) (Table S2). PCR reactions consisted of 0.5 µM of each forward and reverse primer, about 50 ng/µL template DNA, 1× PCR reaction buffer, 400 µM dNTP mix, and 2.5 U G-Taq polymerase (Cosmo, Seoul, Korea). Samples were initially denatured at 94°C for 5 min and then amplified for 35 cycles at 94°C for 45 s, 55°C for 30 s, and 72°C for 90 s. A final extension of 10 min at 72°C was added at the end of the program to ensure complete amplification of the target region. Amplified PCR products were purified using the QIAquick PCR Purification Kit (Qiagen, Valencia, CA, USA). The resulting samples were sent to Macrogen (Seoul, Korea) for pyrosequencing on a 454 Life Sciences Genome Sequencer FLX Titanium machine (Roche). Multiplex pyrosequencing of 68 samples (after filtering) produced 390,444 high-quality 16S rRNA sequences with an average length of 474 bp and 5,741 reads per sample. These sequence data have been submitted to the European Nucleotide Archive under the study number ERP001901.

### Sequence Analysis using QIIME

Data were analyzed using Quantitative Insights Into Microbial Ecology (QIIME) 1.2.0 (http://qiime.sourceforge.net) [31]. Before analyzing the sequences, denoising of the sequence data set was performed [32]. Low-quality sequences were removed (<200 bp), and the 9- or 10-bp barcode was examined to assign sequences to samples. Pyrosequencing data were processed using the QIIME pipeline. Phylotypes were identified using UCLUST and defined at the 97% sequence similarity level. A representative sequence from each phylotype was aligned using PyNAST [33], and the taxonomic identity of each phylotype was determined using the RDP classifier [34]. *Lactobacillus* species level was further identified using the Rtax method with a pre-clustered Greengene database (*gg_97_otus_4feb2011.fasta*) [35]. The differences between each sample pair were determined from a neighbor-joining tree using the weighted UniFrac [36]. Heatmaps of the microbial taxa according to subject were generated by mapping using MultiExperiment Viewer software (version 4.8.01; http://www.tm4.org) [37]. The hierarchical clustering of the bacterial microbiota among subjects was analyzed using Spearman’s correlation.

### Association between Vaginal Microbiota and Phenotype by LEfSe Analysis

To identify a microbiological marker of a vaginal microbiota associated with HPV infection, sequence analyses were performed using a pyrosequencing pipeline with Mothur [38], abundantOTU [39], and PERL scripts. To qualify for analysis, a sequence had to be at least >200 bp or <600 bp in length, have a read quality score >25, and have a barcode match. Each processed sequence was assigned, and the barcode and primer sequences were trimmed. All processed sequences were aligned by the NAST-based sequence aligner to custom references based on the SILVA alignment [38]. The Mothur package of the ChimeraSlayer algorithm [40] was used to identify the chimeric sequences. After sequence identification, sequences were clustered into OTUs using abundantOTU [39]. Representative sequences per OTU were classified using the MSU RDP classifier v2.2 maintained at the Ribosomal Database Project (RDP 10 database, v6). Finally, the abundant OTUs were used in the LEfSe (linear discriminant analysis [LDA] coupled with effect size measurements) to characterize the potential microbial OTU markers with specific disease phenotype [41]. SPSS (ver. 19.0; Armonk, NY, USA) was used to perform either the *t*-test or the Wilcoxon signed-rank test.

## Results and Discussion

### 1) Composition of the Vaginal Microbiota in the Twin Cohort

**Figure 1:**
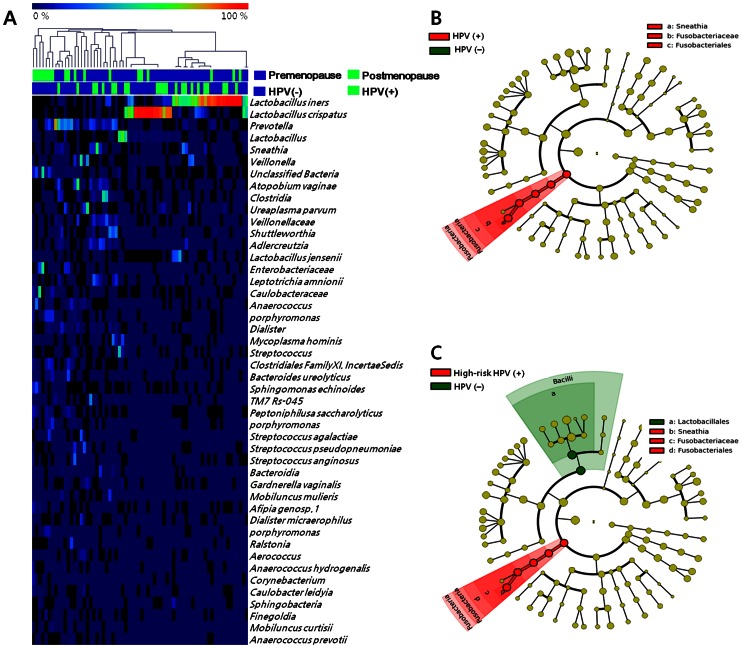
Comparison of the vaginal microbiotas of the study population. (A) Heatmap of abundances of members of the vaginal microbiota; red indicates a high proportion and blue shows a low proportion. (B) Comparison of HPV-negative women (N = 26) and HPV-positive those (N = 19) without CIN. (C) Comparison of HPV-negative (N = 26) and women infected with high-risk HPV types (N = 13) without CIN.

In this study, we investigated the compositional differences between the vaginal microbiota of healthy and HPV-positive individuals using 68 Korean twins and their family members. Vaginal microbiota composition varied significantly among individual subjects (Fig. 1 and Figure S1). The vast majority of the vaginal microbiota belonged to one of six major phyla: Firmicutes, Bacteroidetes, Fusobacteria, Actinobacteria, Tenericutes, and Proteobacteria (Table S3A). Of these, Firmicutes, to which *Lactobacillus* spp. belong, was the most abundant (>77%) in normal premenopausal women [42]. However, in postmenopausal women, the proportion of Firmicutes, including *Lactobacillus* spp., was much lower (<57%), and Proteobacteria were significantly more abundant, including species in the families Enterobacteriaceae and Caulobacteraceae, in addition to *Streptococcus* spp. and *Anaerococcus* spp. (Table S3). These results are consistent with a previous culture-based study reporting a higher proportion of vaginal Proteobacteria (*i.e.*, *Escherichia coli*) in postmenopausal women than premenopausal women [43].

As described above, *Lactobacillus* spp. was the most abundant microorganism. Of the *Lactobacillus* species identified in vaginal microbiotas, *L. iners* and *L. crispatus* were most abundant (29.5% for *L. iners*, and 21.4% for *L. crispatus*) in this study (Fig. 1A, and Figure S1). In addition, *L. jensenii* were identified in only 1.4% of subjects, while no *L. gasseri* were found. These results are in accordance with a previous report of the vaginal microbiomes in reproductive-age Asian women [13]. Specifically, the communities clustered into five groups (clusters I–V). Our subjects who were also Asian, belonged mostly to cluster III (*L. iners*) and cluster I (*L. crispatus*). The prevalence of HPV infection did not differ significantly based on the type of vaginal microbiota (36.6% for cluster III (*L. iners)*, and 21.5% for cluster I (*L. crispatus*) or menopausal status (16.6% for *L. iners*, and 16.2 for *L. crispatus*). The prevalence of HPV infection did not differ between clusters (cluster III and cluster I) of *Lactobacillus* spp. (p = 0.17). Regardless of the *Lactobacillus* spp. cluster, the observed decrease in the prevalence of *Lactobacillus* spp. in subjects that had undergone menopause was consistent with the results of a previous study [44].

We performed LEfSe analysis using both premenopausal healthy women with and without HPV infection (N = 45) to identify a potential microbiological predictor of HPV infection (Fig. 1B) and high-risk HPV (HPV genotype 16 [N = 2], 18 [N = 2], 39 [N = 3], 45 [N = 1], 52 [N = 1], 56 [N = 2], and 59 [N = 2])-infected groups (N = 13) without CIN (Fig. 1C). HPV infection was strongly associated with the abundance of various vaginal microbiota species, particularly Fusobacteria. *Sneathia* spp. were identified as microbiological markers of HPV infection (Fig. 1C and Table S3B). Additionally, when we further analyzed the vaginal microbiota based on HPV virulence type, *Sneathia* was identified as a microbiological marker of high-risk HPV infection (Fig. 1C and Table S3B). *Sneathia* was the most abundant genus, being more than three times more frequent among women in the high-risk HPV-infected group.

**Figure 2:**
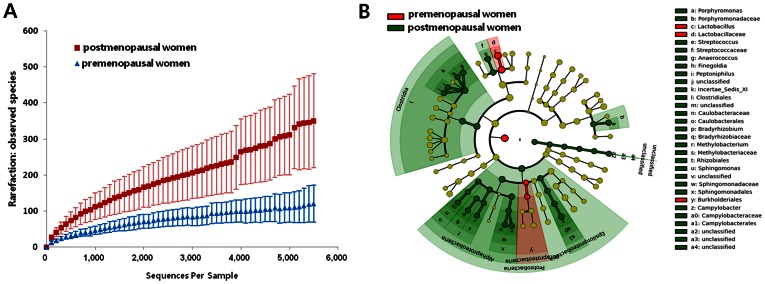
The effect of menopause on the vaginal microbiota. (A) Rarefaction curves for postmenopausal women (N = 18) and premenopausal women without either HPV infection or CIN (N = 26). (B) Microbiological markers of postmenopausal (N = 18) and premenopausal women without either HPV infection or CIN (N = 26) by LEfSe.

Overall, the microbial species richness, which is directly associated with the microbial diversity of postmenopausal women, was significantly higher than that of premenopausal women, as indicated by the rarefaction curves in Figure 2A. These results are consistent with a previous report that bacterial diversity is higher in menopausal women [44]. To identify the specific taxa differentially present or abundant between these microbiomes, we employed the LEfSe method [45]. As expected, a higher *Lactobacillus* spp. abundance was strongly associated with premenopausal women (Fig. 2B). Diverse microorganisms, including species of the genera *Porphyromonas*, *Streptococcus*, *Sphingomonas*, *Camplylobacter*, and *Peptoniphilus*, were significantly associated with postmenopausal women (Fig. 2B and Table S3B). These results are consistent with a previous report of *Streptococcus* abundance in postmenopausal women [46]. Vaginal microbiotas are influenced by estrogen levels, which also control the thickness of the vaginal epithelial tissue. Therefore, epithelial thinning in postmenopausal women could be responsible for the change in the distribution of the vaginal microbiota [46]. Despite the small sample size, our results clearly indicate that changes in the vaginal microbiota after menopause are attributable mostly to hormonal changes. Our preliminary results suggest that the vaginal microbiotas of postmenopausal women receiving HRT decreased dramatically and were similar to those of premenopausal women (Figure S2). Despite the small sample size in our study, these results are consistent with previous reports that postmenopausal women receiving HRT had a lower vaginal pH and a lower microbial richness than women not receiving HRT [47].

Because menopause status was strongly-associated with the vaginal microbiota, samples from postmenopausal women were excluded from the investigation of the association between the vaginal microbiota and HPV infection. After excluding the 18 postmenopausal women, we compared the vaginal microbiota of 23 HPV-positive and 27 HPV-negative premenopausal women. Among the 23 HPV-positive premenopausal women, four had CIN, which is a potential precursor of cervical cancer. In contrast, only one had CIN among the 27 HPV-negative premenopausal women. The average abundance of *Lactobacillus* spp. was much lower in groups infected with HPV or with CIN than in the premenopausal control group. In the CIN groups, the proportions of Lachnospiraceae and *Veillonella* spp. became higher (Table S3). Instead, Fusobacteria were more prevalent in the HPV-infected group than in the uninfected or postmenopausal group (*P*<0.05, *t*-test) (Fig. 1B and Table S3). Other microorganisms including *Prevotella* spp. (phylum Bacteroidetes), *Sneathia* spp. (phylum Fusobacteria), and *Clostridiales* (phylum Firmicutes), significantly increased among the group with HPV infection. These results indicate that HPV infection was significantly associated with the composition of the vaginal microbiota.

### 2) Comparison of Microbiota in HPV-infected Discordant MZ Twins

**Figure 3:**
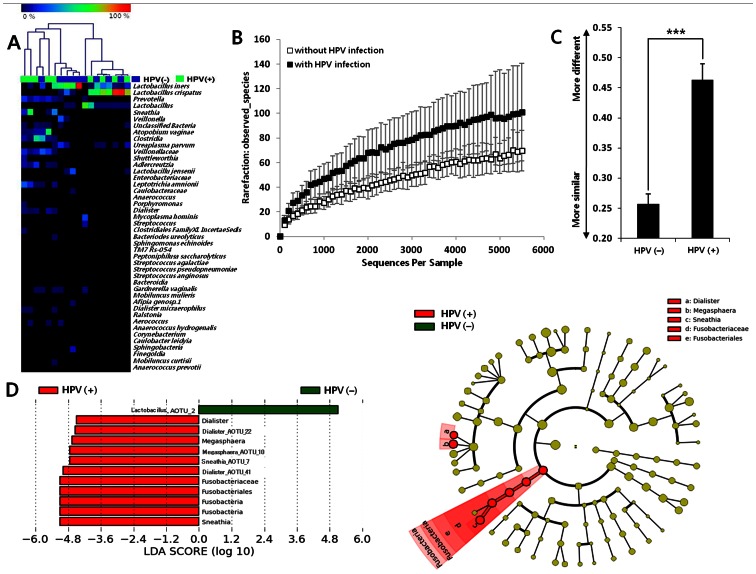
A comparison of the vaginal microbiota among nine HPV infection-discordant twin pairs (N = 18). (A) Heatmap of the vaginal microbiota at the species levels of HPV infection-discordant MZ twin pairs (N = 9 each for HPV [+] and [−]). (B) Rarefaction curve for the vaginal microbiota (mean ±95% confidence interval) of nine HPV infection-discordant MZ twin pairs (N = 18). (C) Average weighted UniFrac distance between the discordant MZ twin pairs (nine pairs [N = 18]) (***P<10^−8^). (D) HPV-negative and HPV-positive women in nine MZ HPV-discordant twin pairs (N = 18).

To adjust for potential genetic confounding factors, we characterized the association between HPV infection and the vaginal microbiota using nine HPV infection-discordant MZ twin pairs without CIN (N = 18) (Fig. 3). Despite the small sample size, the data for the nine discordant twin pairs showed clear differences in the vaginal microbiota between HPV-positive and negative subjects. The occurrence of HPV infection was strongly associated with a decrease in *Lactobacillus* spp. and other facultative or anaerobic species. The percentage of *Lactobacillus* spp. was significantly lower in the HPV-infected group (mean 47%) than in the uninfected group (mean 77%). In particular, a decrease in the abundance of *L. iners* in the vaginal microbiota was associated with HPV infection among discordant MZ twins (*P* = 0.03) (Fig. 3A and Table S3). However, the abundance of *L. crispatus* was not significantly associated with HPV infection (*P* = 0.76). *Lactobacillus* spp. are prevalent in the vagina, where they play a role in maintaining a low pH through their metabolic activities. Thus, *Lactobacillus* spp. might confer resistance to HPV infection in addition to protecting against colonization of overt pathogens or against dominance by potentially pathogenic species [42], [48]. For example, the E5 protein of HPV type 16 is quite susceptible to low pH [49].

In the present study, higher proportions of *Prevotella*, *Sneathia*, *Dialister,* and *Bacillus* species were detected in the HPV-infected than in HPV-negative discordant MZ twin pairs (Table S3). *Prevotella* and *Sneathia* were previously found in BV-positive women [10]. *Dialister* spp. are more frequent in women of reproductive age with a high vaginal pH [13]. The roles of these microorganisms are not clear and should be investigated further. The vaginal microbiota of the HPV-positive group was markedly more diverse than that of the HPV-negative group (Fig. 3B). Increased microbial richness (a component of species diversity) with HPV infection was also confirmed by the average weighted UniFrac distance between the HPV-positive twins and the negative-discordant control (*P*<10^−8^; Fig. 3C). These results suggest that the vaginal microbiota differed significantly according to HPV infection status, even after adjusting for genetic factors within the MZ twins.

We performed LEfSe analysis using HPV-discordant twin pairs (N = 18) (Fig. 3D). *Sneathia* and *Megasphaera* were significantly associated with HPV infection. *Sneathia* spp. were identified in a previous study of BV-positive women, although their HPV infection status was unknown [1]. Species in this genus, which are lactic acid producers like those in *Lactobacillus*, may represent useful microbiological predictors of HPV infection among healthy premenopausal women. However, in this study, the LEfSe analysis indicated only differences in operational taxonomic units (OTUs) and did not detect the presence or absence of *Sneathia* spp. (Fig. 3D). Therefore, the specificity of *Sneathia* spp. as a potential predictor of HPV infection should be evaluated further. Fusobacteria, including *Fusobacterium* and *Sneathia* spp., typically inhabit mammalian mucous membranes and can invade epithelial cells; these pathogens thus cause a wide range of human infections [41], [50]. Adherence to epithelial cells is crucial for colonization, and invasion allows the bacteria to evade the host immune surveillance and spread into the deeper tissues [51]. These events could elicit host pro-inflammatory responses [52] and expression of virulence characteristics, which could further promote the adhesiveness of Fusobacteria to host epithelial cells [53], [54]. A previous study reported that *Fusobacterium* spp. may be associated with inflammatory bowel disease (IBD) [55]. Recently, Kostic *et al*. reported the association of *Fusobacterium* with colorectal carcinoma [41]. The results of our study are consistent with these studies, and suggest strongly that Fusobacteria is a key dysbiosis-associated microorganism that induces an imbalance in mucosal immunity and subsequent adverse health effects. In this study, we used two different bioinformatics tools (QIIME and LEfSe) to analyze the vaginal microbiota; QIIME was used to compare the entire microbial structure within the study subpopulation, while LEfSe was applied to identify specific microbiological associations for disease phenotypes. The results of both analyses clearly suggest a strong association between HPV infection and the composition of the vaginal microbiota.

It should be noted that our study demonstrates only an association between the composition of the vaginal microbiota and HPV infection. The possibility exists that a lower proportion of *Lactobacillus* spp. could render the vaginal environment more susceptible to HPV infection. Previous studies have indicated that BV is significantly associated with sexually transmitted viral diseases, including HPV [56], [57]. However, HPV infection may alter mucosal metabolism [46], host immunity [58], or both, resulting in changes in the community structure of vaginal microbiota. For example, HPVs infect stratified basal vaginal epithelial cells, and viral particles infect the host by entering the basal epithelial cells through a break in the skin [23]. When the vaginal epithelium becomes thin, the levels of glycogen would be greatly reduced. Because glycogen is typically metabolized to lactic acid by *Lactobacillus* spp. in the vaginal environment, the reduction in glycogen level is at least partially responsible for the changes in vaginal pH, which lead to changes in the composition of the microbiota [46]. Several previous studies have found that HIV infection is associated with a higher vaginal microbiota diversity [10], [14]. HPV is known to infect vaginal mucosal surfaces [59], [60]; mucosal immunity and inflammation typically occur during virus infection [58], mediated by several mechanisms including the induction of interferon and activation of macrophages and NK cells. Pro-inflammatory cytokines, reactive oxygen species, viral DNA integration, and chronic inflammation during HPV infection could also result in changes in the vaginal mucosal environment, leading to changes in the vaginal microbiota [60], [61], [62]. Future studies are necessary to elucidate the causality and mechanisms of the associations between HPV infection and changes in vaginal microbiota.

### 3) Comparison of Vaginal Microbiota of MZ Twins and their Families

**Figure 4:**
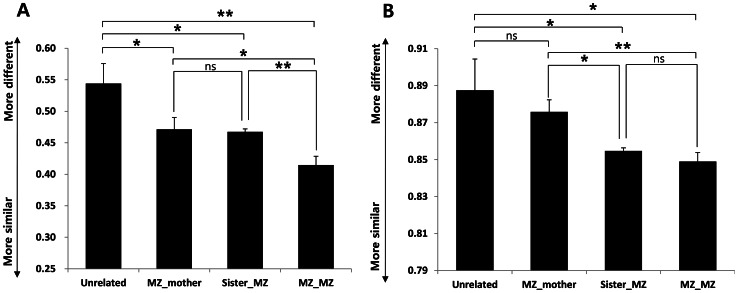
Average weighted (A) and unweighted (B) UniFrac distances between twin pairs, twins and mothers, and unrelated individuals (Student’s *t*-test; mean ± SEM; *P<0.05, **P<10^−4^,; MZ = 13 pairs (N = 26), MZ’s mother = 8, MZ’s sister = 8).

Vaginal microbiota from twins, siblings, and their mothers were compared using both weighted and unweighted UniFrac distances (Fig. 4A and 4B). Based on both average weighted and unweighted UniFrac distances (Fig. 4A and 4B), the vaginal microbiotas of MZ twins were significantly more similar to each other than to the microbiotas of unrelated individuals (*P*<0.05). Both average weighted and unweighted UniFrac distances of vaginal microbiotas were most similar between MZ and MZ, followed by MZ-sister, MZ-mother, and then among unrelated subjects. These results suggest associations between genetics and the vaginal microbiota. The similarity of the vaginal microbiotas of twins was due to both shared OTUs (unweighted) and shared OTU population distributions (weighted). In addition, the vaginal microbiotas of MZ twins were much more similar than to those of their sisters and mothers (*P*<0.05). The distributions of shared OTUs of the vaginal microbiotas of twins were significantly different from those of their sisters (*P*<10^−4^). However, shared OTUs did not differ significantly according to unweighted UniFrac results (*P*>0.05). The microbiotas of MZ twins were significantly different from those of their mothers when analyzed by both weighted (*P*<0.05) and unweighted UniFrac (*P*<10^−4^). All mothers were 55−73 years old and menopausal (Table S1). Many factors differed between the twins and their parents [24]. As described above, the menopausal status resulted in significant differences in vaginal microbiota within the same family. These results suggest strongly that unique genetic, physiological, and environmental factors within the family contribute significantly to the unique presence and distribution of the vaginal microbiota.

### Conclusion

This study provides strong evidence that an increased vaginal microbiota diversity is strongly associated with HPV infection. In particular, Fusobacteria, including *Sneathia* spp., were strongly associated with HPV infection. In addition, both the composition and diversity of the vaginal microbiota were influenced by host genetics, physiology, and other factors, such as menopause and estrogen level. Potential microbiological predictors identified from this study may provide insight into the pathogenesis of HPV and may represent potential microbiological targets for novel diagnostic methodologies that will improve women’s health status.

## Supporting Information

Figure S1 **Relative abundances of microbiota among populations with/without HPV infection and having undergone menopause.** Numbers are the numbers in accordance with those shown in Table S1.
(TIF)

Figure S2 (A) Rarefaction curves for postmenopausal women receiving HRT (N = 3) and not receiving HRT (N = 15). (B) Microbiological markers of postmenopausal (N = 18) and premenopausal women without either HPV infection or CIN (N = 26) by LEfSe. (C) LDA scores and markers of postmenopausal women receiving (N = 3) and not receiving HRT (N = 15) by LEfSe.
(TIF)

Table S1 Summary of the epidemiological and clinical information of this study population (N = 68).
(DOC)

Table S2 Barcoded universal primers for amplification of 16S rRNA.
(DOC)

Table S3 Summary of vaginal microbiota phyla (A) and the relative abundance of the top 50 most abundant genera (B) and species (C) (Average ± SD).
(DOC)
