# Supplementary figures and images for: Association of the Vaginal Microbiota with Human Papillomavirus Infection in a Korean Twin Cohort
Source: PLoS One. 2013 May 22;8(5):e63514. doi: 10.1371/journal.pone.0063514 (PMC3661536; doi:10.1371/journal.pone.0063514)

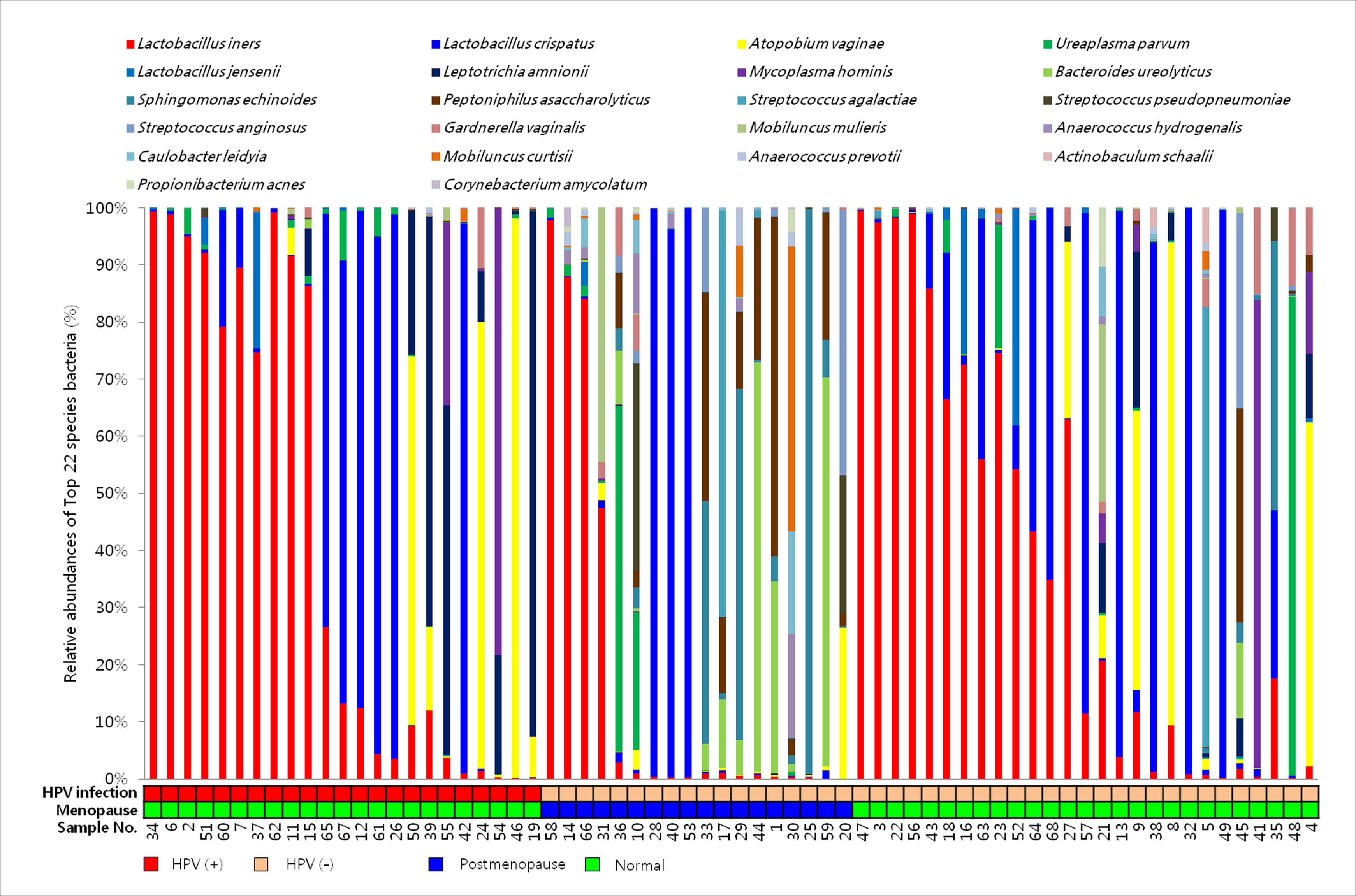

Supplement: Figure S1 — Relative abundances of microbiota among populations with/without HPV infection and having undergone menopause. Numbers are the numbers in accordance with those shown in Table S1. (TIF) [file pone.0063514.s001.tif]

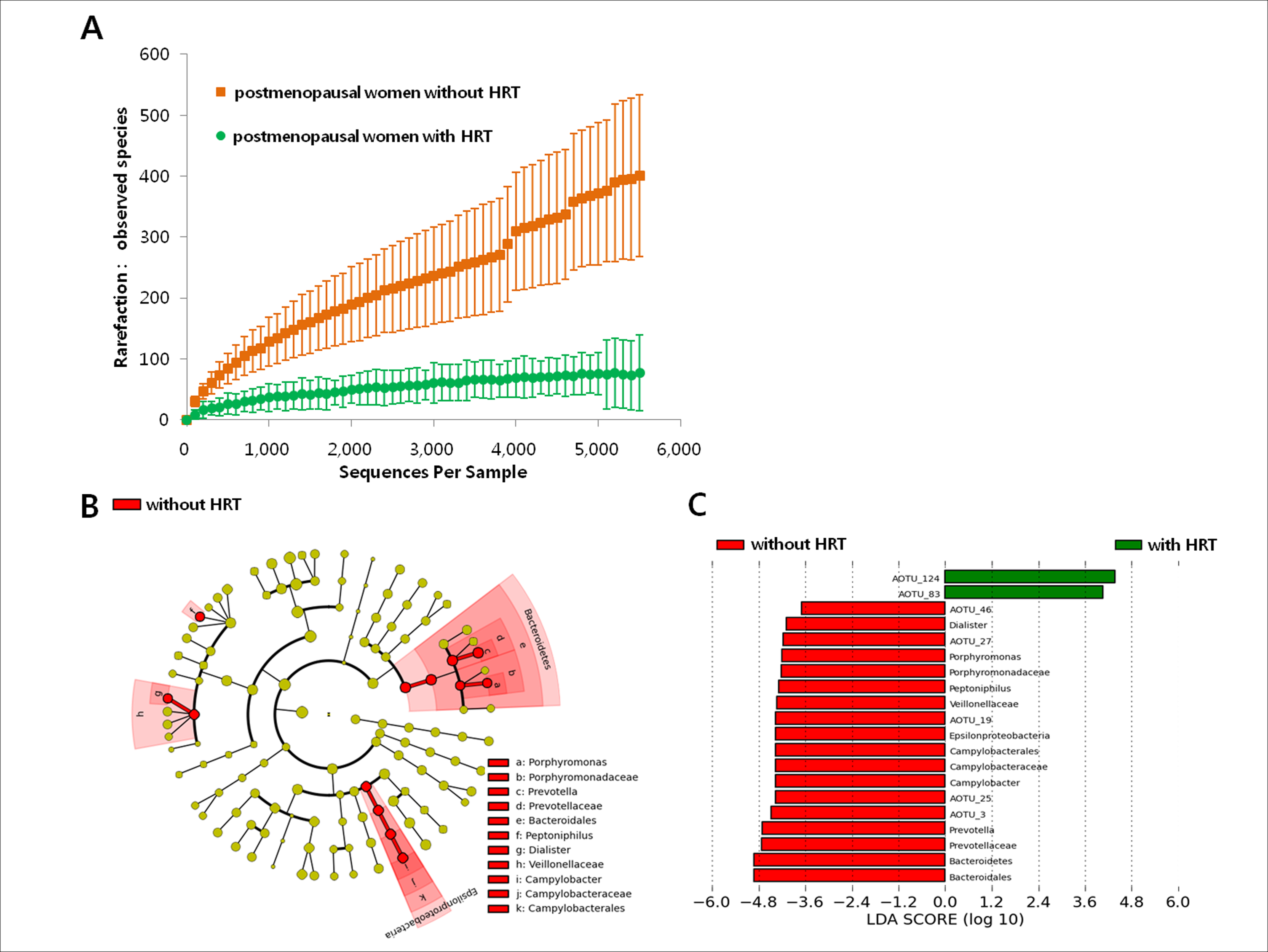

Supplement: Figure S2 — (A) Rarefaction curves for postmenopausal women receiving HRT (N = 3) and not receiving HRT (N = 15). (B) Microbiological markers of postmenopausal (N = 18) and premenopausal women without either HPV infection or CIN (N = 26) by LEfSe. (C) LDA scores and markers of postmenopausal women receiving (N = 3) and not receiving HRT (N = 15) by LEfSe. (TIF) [file pone.0063514.s002.tif]
