# Supplementary material for: Association of the Vaginal Microbiota with Human Papillomavirus Infection in a Korean Twin Cohort
Source: PLoS One. 2013 May 22;8(5):e63514. doi: 10.1371/journal.pone.0063514 (PMC3661536; doi:10.1371/journal.pone.0063514)
Supplement: Table S1 — Summary of the epidemiological and clinical information of this study population (N = 68). (DOC) [file pone.0063514.s003.doc]

**Table S1:** Summary of epidemiological and clinical information of this study population (N=68).

| **Sample** | **Family ID** | **Age (yr)** | **Menopause** | **HRT^*^** | **Clinical test**^†^ | **Zygosity^‡^** | **HPV infection^§^** | **HPV genotype** |
| --- | --- | --- | --- | --- | --- | --- | --- | --- |
| 1 | 1000053 | 71 | yes | no | normal | mother | HPV neg. |  |
| 2 | 1000053 | 43 | no |  | normal | DZ **^**^** | High | 39 |
| 3 | 1000053 | 43 | no |  | normal | DZ **^**^** | HPV neg. |  |
| 4 | 1000098 | 35 | no |  | normal | MZ **^**^** | HPV neg. |  |
| 5 | 1000098 | 35 | no |  | CIN | MZ **^**^** | HPV neg. |  |
| 6 | 1000102 | 47 | no |  | CIN | MZ **^#^** | High | 59 |
| 7 | 1000128 | 48 | no |  | normal | sibling | High | 56 |
| 8 | 1000128 | 45 | no |  | normal | sibling | HPV neg. |  |
| 9 | 1000128 | 42 | no |  | normal | sibling | HPV neg. |  |
| 10 | 1000135 | 63 | yes | no | normal | mother | HPV neg. |  |
| 11 | 1000135 | 40 | no |  | normal | sibling | High | 52 |
| 12 | 1000142 | 42 | no |  | CIN | sibling | High | 16 |
| 13 | 1000142 | 47 | no |  | normal | MZ **^#^** | HPV neg. |  |
| 14 | 1000152 | 50 | yes | no | normal | MZ **^#^** | HPV neg. |  |
| 15 | 1000182 | 48 | no |  | normal | MZ **^¶ **^** | Low | 70 |
| 16 | 1000182 | 48 | no |  | normal | MZ **^¶ **^** | HPV neg. |  |
| 17 | 1000195 | 66 | yes | no | normal | mother | HPV neg. |  |
| 18 | 1000195 | 42 | no |  | normal | MZ **^¶ **^** | HPV neg. |  |
| 19 | 1000195 | 42 | no |  | normal | MZ **^¶ **^** | High | 39 |
| 20 | 1000196 | 66 | yes | no | normal | mother | HPV neg. |  |
| 21 | 1000196 | 40 | no |  | normal | sibling | HPV neg. |  |
| 22 | 1000196 | 34 | no |  | normal | sibling | HPV neg. |  |
| 23 | 1000196 | 31 | no |  | normal | MZ **^¶ **^** | HPV neg. |  |
| 24 | 1000196 | 31 | no |  | normal | MZ **^¶ **^** | High | 18 |
| 25 | 1000215 | 58 | yes | yes | normal | mother | HPV neg. |  |
| 26 | 1000215 | 35 | no |  | normal | MZ **^¶ **^** | High | 16 |
| 27 | 1000215 | 35 | no |  | normal | MZ **^¶ **^** | HPV neg. |  |
| 28 | 1000219 | 49 | yes | yes | normal | MZ **^#^** | HPV neg. |  |
| 29 | 1000285 | 73 | yes | no | normal | MZ **^#^** | HPV neg. |  |
| 30 | 1000297 | 60 | yes | no | normal | sibling | HPV neg. |  |
| 31 | 1000297 | 54 | yes | no | normal | sibling | HPV neg. |  |
| 32 | 1000306 | 36 | no |  | normal | MZ **^#^** | HPV neg. |  |
| 33 | 1000312 | 66 | yes | no | normal | mother | HPV neg. |  |
| 34 | 1000312 | 46 | no |  | CIN | MZ **^**^** | High | 56 |
| 35 | 1000312 | 46 | no |  | normal | MZ **^**^** | HPV neg. |  |
| 36 | 1000313 | 61 | yes | no | normal | mother | HPV neg. |  |
| 37 | 1000313 | 37 | no |  | normal | MZ **^#^** | probably high | 66 |
| 38 | 1000338 | 40 | no |  | normal | MZ **^¶ **^** | HPV neg. |  |
| 39 | 1000338 | 40 | no |  | normal | MZ **^¶ **^** | High | 45 |
| 40 | 1000372 | 55 | yes | yes | normal | mother | HPV neg. |  |
| 41 | 1000372 | 35 | no |  | normal | MZ **^¶ **^** | HPV neg. |  |
| 42 | 1000372 | 35 | no |  | normal | MZ **^¶ **^** | High | 39 |
| 43 | 1000372 | 31 | no |  | normal | sibling | HPV neg. |  |
| 44 | 1000376 | 73 | yes | no | normal | mother | HPV neg. |  |
| 45 | 1000376 | 52 | no |  | normal | sibling | HPV neg. |  |
| 46 | 1000376 | 49 | no |  | normal | MZ **^¶ **^** | undetermined | 67 |
| 47 | 1000376 | 49 | no |  | normal | MZ **^¶ **^** | HPV neg. |  |
| 48 | 1000376 | 47 | no |  | normal | sibling | HPV neg. |  |
| 49 | 1000391 | 32 | no |  | normal | MZ **^#^** | HPV neg. |  |
| 50 | 1000396 | 34 | no |  | normal | MZ **^**^** | High | 56 |
| 51 | 1000396 | 34 | no |  | normal | MZ **^**^** | High | 52 |
| 52 | 2000013 | 52 | no |  | normal | sibling | HPV neg. |  |
| 53 | 2000013 | 49 | yes | no | normal | DZ **^**^** | HPV neg. |  |
| 54 | 2000013 | 49 | no |  | normal | DZ **^**^** | High | 18 |
| 55 | 2000025 | 44 | no |  | CIN | sibling | undetermined | 90 |
| 56 | 2000025 | 41 | no |  | normal | sibling | HPV neg. |  |
| 57 | 2000079 | 47 | no |  | normal | sibling | HPV neg. |  |
| 58 | 2000079 | 51 | yes | no | normal | sibling | HPV neg. |  |
| 59 | 2000088 | 73 | yes | no | normal | mother | HPV neg. |  |
| 60 | 2000088 | 42 | no |  | normal | MZ **^**^** | Low | 70 |
| 61 | 2000088 | 42 | no |  | normal | MZ **^**^** | Low | 70 |
| 62 | 2000116 | 36 | no |  | normal | MZ **^#^** | High | 16 |
| 63 | 2000117 | 37 | no |  | normal | MZ **^¶ **^** | HPV neg. |  |
| 64 | 2000117 | 37 | no |  | normal | MZ **^¶ **^** | undetermined | 74 |
| 65 | 2000117 | 43 | no |  | normal | sibling | HPV neg. |  |
| 66 | 3000014 | 58 | yes | no | normal | sibling | HPV neg. |  |
| 67 | 3000014 | 45 | no |  | normal | MZ **^¶ **^** | High | 59 |
| 68 | 3000014 | 45 | no |  | normal | MZ **^¶ **^** | HPV neg. |  |

^*^HRT: hormone replacement therapy

^†^CIN: cervical intraepithelial neoplasia

^‡^MZ: monozygotic twin; DZ: dizygotic twin

^§^High: high-risk types of HPV; Low: low-risk types of HPV

^¶^HPV-discordant MZ; 9 pairs without CIN

^#^Unpaired MZ pairs (N=9)

^**^Paired MZ twin (N=26 (13 pairs))
