# Supplementary material for: Association of the Vaginal Microbiota with Human Papillomavirus Infection in a Korean Twin Cohort
Source: PLoS One. 2013 May 22;8(5):e63514. doi: 10.1371/journal.pone.0063514 (PMC3661536; doi:10.1371/journal.pone.0063514)
Supplement: Table S2 — Barcoded universal primers for amplification of 16S rRNA. (DOC) [file pone.0063514.s004.doc]

**Table S2: Barcoded universal primers for amplification of 16S rRNA.**

| **Primer name** | | **454 B adapter sequence** | **Linker** | | **Forward 16S rRNA sequence** |
| --- | --- | --- | --- | --- | --- |
| 454_8F | | CCTATCCCCTGTGTGCCTTGGCAGTC | TCAG | | AGAGTTTGATCCTGGCTCAG |
|  |  |  |  |  |  |
| **Primer name** | **Barcode ID** | **454 A adapter sequence** | **Linker** | **Barcode sequence** | **Reverse 16S rRNA sequence** |
| 454_534R_1 | MID1 | CCATCTCATCCCTGCGTGTCTCCGAC | TCAG | ACGAGTGCGT | ATTACCGCGGCTGCTGG |
| 454_534R_2 | MID2 | CCATCTCATCCCTGCGTGTCTCCGAC | TCAG | ACGCTCGACA | ATTACCGCGGCTGCTGG |
| 454_534R_3 | MID3 | CCATCTCATCCCTGCGTGTCTCCGAC | TCAG | AGACGCACTC | ATTACCGCGGCTGCTGG |
| 454_534R_4 | MID4 | CCATCTCATCCCTGCGTGTCTCCGAC | TCAG | ATATCGCGAG | ATTACCGCGGCTGCTGG |
| 454_534R_5 | MID5 | CCATCTCATCCCTGCGTGTCTCCGAC | TCAG | CGTGTCTCTA | ATTACCGCGGCTGCTGG |
| 454_534R_6 | MID6 | CCATCTCATCCCTGCGTGTCTCCGAC | TCAG | TAGCTCTATC | ATTACCGCGGCTGCTGG |
| 454_534R_7 | MID7 | CCATCTCATCCCTGCGTGTCTCCGAC | TCAG | TGATACGTCT | ATTACCGCGGCTGCTGG |
| 454_534R_8 | MID8 | CCATCTCATCCCTGCGTGTCTCCGAC | TCAG | TATAGACATC | ATTACCGCGGCTGCTGG |
| 454_534R_9 | MID9 | CCATCTCATCCCTGCGTGTCTCCGAC | TCAG | CATAGTAGTG | ATTACCGCGGCTGCTGG |
| 454_534R_10 | MID10 | CCATCTCATCCCTGCGTGTCTCCGAC | TCAG | TCACGTACTA | ATTACCGCGGCTGCTGG |
| 454_534R_11 | MID11 | CCATCTCATCCCTGCGTGTCTCCGAC | TCAG | CGTCTAGTAC | ATTACCGCGGCTGCTGG |
| 454_534R_12 | MID12 | CCATCTCATCCCTGCGTGTCTCCGAC | TCAG | ACGACTACAG | ATTACCGCGGCTGCTGG |
| 454_534R_13 | MID13 | CCATCTCATCCCTGCGTGTCTCCGAC | TCAG | CGTAGACTAG | ATTACCGCGGCTGCTGG |
| 454_534R_14 | MID14 | CCATCTCATCCCTGCGTGTCTCCGAC | TCAG | TACTCTCGTG | ATTACCGCGGCTGCTGG |
| 454_534R_15 | MID15 | CCATCTCATCCCTGCGTGTCTCCGAC | TCAG | TAGAGACGAG | ATTACCGCGGCTGCTGG |
| 454_534R_16 | MID16 | CCATCTCATCCCTGCGTGTCTCCGAC | TCAG | TCGTCGCTCG | ATTACCGCGGCTGCTGG |
| 454_534R_17 | MID17 | CCATCTCATCCCTGCGTGTCTCCGAC | TCAG | ACATACGCGT | ATTACCGCGGCTGCTGG |
| 454_534R_18 | MID18 | CCATCTCATCCCTGCGTGTCTCCGAC | TCAG | ACGCGAGTAT | ATTACCGCGGCTGCTGG |
| 454_534R_19 | MID19 | CCATCTCATCCCTGCGTGTCTCCGAC | TCAG | ACTACTATGT | ATTACCGCGGCTGCTGG |
| 454_534R_20 | MID20 | CCATCTCATCCCTGCGTGTCTCCGAC | TCAG | ACTGTACAGT | ATTACCGCGGCTGCTGG |
| 454_534R_21 | MID21 | CCATCTCATCCCTGCGTGTCTCCGAC | TCAG | AGACTATACT | ATTACCGCGGCTGCTGG |
| 454_534R_22 | MID22 | CCATCTCATCCCTGCGTGTCTCCGAC | TCAG | AGCGTCGTCT | ATTACCGCGGCTGCTGG |
| 454_534R_23 | MID23 | CCATCTCATCCCTGCGTGTCTCCGAC | TCAG | AGTACGCTAT | ATTACCGCGGCTGCTGG |
| 454_534R_24 | MID24 | CCATCTCATCCCTGCGTGTCTCCGAC | TCAG | ATAGAGTACT | ATTACCGCGGCTGCTGG |
| 454_534R_25 | MID25 | CCATCTCATCCCTGCGTGTCTCCGAC | TCAG | CACGCTACGT | ATTACCGCGGCTGCTGG |
| 454_534R_26 | MID26 | CCATCTCATCCCTGCGTGTCTCCGAC | TCAG | CAGTAGACGT | ATTACCGCGGCTGCTGG |
| 454_534R_27 | MID27 | CCATCTCATCCCTGCGTGTCTCCGAC | TCAG | CGACGTGACT | ATTACCGCGGCTGCTGG |
| 454_534R_28 | MID28 | CCATCTCATCCCTGCGTGTCTCCGAC | TCAG | TACACGTGAT | ATTACCGCGGCTGCTGG |
| 454_534R_29 | MID29 | CCATCTCATCCCTGCGTGTCTCCGAC | TCAG | TACGCTGTCT | ATTACCGCGGCTGCTGG |
| 454_534R_30 | MID30 | CCATCTCATCCCTGCGTGTCTCCGAC | TCAG | TCGATCACGT | ATTACCGCGGCTGCTGG |
| 454_534R_31 | MID31 | CCATCTCATCCCTGCGTGTCTCCGAC | TCAG | TCGCACTAGT | ATTACCGCGGCTGCTGG |
| 454_534R_32 | MID32 | CCATCTCATCCCTGCGTGTCTCCGAC | TCAG | TGACGTATGT | ATTACCGCGGCTGCTGG |
| 454_534R_33 | MID33 | CCATCTCATCCCTGCGTGTCTCCGAC | TCAG | ACAGTATATA | ATTACCGCGGCTGCTGG |
| 454_534R_34 | MID34 | CCATCTCATCCCTGCGTGTCTCCGAC | TCAG | ACGCGATCGA | ATTACCGCGGCTGCTGG |
| 454_534R_35 | MID35 | CCATCTCATCCCTGCGTGTCTCCGAC | TCAG | ACTAGCAGTA | ATTACCGCGGCTGCTGG |
| 454_534R_36 | MID36 | CCATCTCATCCCTGCGTGTCTCCGAC | TCAG | AGCTCACGTA | ATTACCGCGGCTGCTGG |
| 454_534R_37 | MID37 | CCATCTCATCCCTGCGTGTCTCCGAC | TCAG | AGTATACATA | ATTACCGCGGCTGCTGG |
| 454_534R_38 | MID38 | CCATCTCATCCCTGCGTGTCTCCGAC | TCAG | AGTGCTACGA | ATTACCGCGGCTGCTGG |
| 454_534R_39 | MID39 | CCATCTCATCCCTGCGTGTCTCCGAC | TCAG | CGATCGTATA | ATTACCGCGGCTGCTGG |
| 454_534R_40 | MID40 | CCATCTCATCCCTGCGTGTCTCCGAC | TCAG | CGCAGTACGA | ATTACCGCGGCTGCTGG |
| 454_534R_41 | MID41 | CCATCTCATCCCTGCGTGTCTCCGAC | TCAG | CGCGTATACA | ATTACCGCGGCTGCTGG |
| 454_534R_42 | MID42 | CCATCTCATCCCTGCGTGTCTCCGAC | TCAG | CGTACAGTCA | ATTACCGCGGCTGCTGG |
| 454_534R_43 | MID43 | CCATCTCATCCCTGCGTGTCTCCGAC | TCAG | CGTACTCAGA | ATTACCGCGGCTGCTGG |
| 454_534R_44 | MID44 | CCATCTCATCCCTGCGTGTCTCCGAC | TCAG | CTACGCTCTA | ATTACCGCGGCTGCTGG |
| 454_534R_45 | MID45 | CCATCTCATCCCTGCGTGTCTCCGAC | TCAG | CTATAGCGTA | ATTACCGCGGCTGCTGG |
| 454_534R_46 | MID46 | CCATCTCATCCCTGCGTGTCTCCGAC | TCAG | TACGTCATCA | ATTACCGCGGCTGCTGG |
| 454_534R_47 | MID47 | CCATCTCATCCCTGCGTGTCTCCGAC | TCAG | TAGTCGCATA | ATTACCGCGGCTGCTGG |
| 454_534R_48 | MID48 | CCATCTCATCCCTGCGTGTCTCCGAC | TCAG | ATATATACA | ATTACCGCGGCTGCTGG |
| 454_534R_49 | MID49 | CCATCTCATCCCTGCGTGTCTCCGAC | TCAG | CACGCGAGA | ATTACCGCGGCTGCTGG |
| 454_534R_50 | MID50 | CCATCTCATCCCTGCGTGTCTCCGAC | TCAG | TCGATAGTGA | ATTACCGCGGCTGCTGG |
| 454_534R_51 | MID51 | CCATCTCATCCCTGCGTGTCTCCGAC | TCAG | TCGCTGCGTA | ATTACCGCGGCTGCTGG |
| 454_534R_52 | MID52 | CCATCTCATCCCTGCGTGTCTCCGAC | TCAG | TCTGACGTCA | ATTACCGCGGCTGCTGG |
| 454_534R_53 | MID53 | CCATCTCATCCCTGCGTGTCTCCGAC | TCAG | TGAGTCAGTA | ATTACCGCGGCTGCTGG |
| 454_534R_54 | MID54 | CCATCTCATCCCTGCGTGTCTCCGAC | TCAG | TGTAGTGTGA | ATTACCGCGGCTGCTGG |
| 454_534R_55 | MID55 | CCATCTCATCCCTGCGTGTCTCCGAC | TCAG | ACACATACGC | ATTACCGCGGCTGCTGG |
| 454_534R_56 | MID56 | CCATCTCATCCCTGCGTGTCTCCGAC | TCAG | ACAGTCGTGC | ATTACCGCGGCTGCTGG |
| 454_534R_57 | MID57 | CCATCTCATCCCTGCGTGTCTCCGAC | TCAG | ACATGACGAC | ATTACCGCGGCTGCTGG |
| 454_534R_58 | MID58 | CCATCTCATCCCTGCGTGTCTCCGAC | TCAG | ACGTCTCATC | ATTACCGCGGCTGCTGG |
| 454_534R_59 | MID59 | CCATCTCATCCCTGCGTGTCTCCGAC | TCAG | AGAGCGTCAC | ATTACCGCGGCTGCTGG |
| 454_534R_60 | MID60 | CCATCTCATCCCTGCGTGTCTCCGAC | TCAG | AGCGACTAGC | ATTACCGCGGCTGCTGG |
| 454_534R_61 | MID61 | CCATCTCATCCCTGCGTGTCTCCGAC | TCAG | AGTAGTGATC | ATTACCGCGGCTGCTGG |
| 454_534R_62 | MID62 | CCATCTCATCCCTGCGTGTCTCCGAC | TCAG | ATAGATAGAC | ATTACCGCGGCTGCTGG |
| 454_534R_63 | MID63 | CCATCTCATCCCTGCGTGTCTCCGAC | TCAG | ATATAGTCGC | ATTACCGCGGCTGCTGG |
| 454_534R_64 | MID64 | CCATCTCATCCCTGCGTGTCTCCGAC | TCAG | ATCTACTGAC | ATTACCGCGGCTGCTGG |
| 454_534R_65 | MID65 | CCATCTCATCCCTGCGTGTCTCCGAC | TCAG | CACGTAGATC | ATTACCGCGGCTGCTGG |
| 454_534R_66 | MID66 | CCATCTCATCCCTGCGTGTCTCCGAC | TCAG | CGACACTATC | ATTACCGCGGCTGCTGG |
| 454_534R_67 | MID67 | CCATCTCATCCCTGCGTGTCTCCGAC | TCAG | CATACTCTAC | ATTACCGCGGCTGCTGG |
| 454_534R_68 | MID68 | CCATCTCATCCCTGCGTGTCTCCGAC | TCAG | CGAGACGCGC | ATTACCGCGGCTGCTGG |
