# Supplementary material for: Association of the Vaginal Microbiota with Human Papillomavirus Infection in a Korean Twin Cohort
Source: PLoS One. 2013 May 22;8(5):e63514. doi: 10.1371/journal.pone.0063514 (PMC3661536; doi:10.1371/journal.pone.0063514)
Supplement: Table S3 — Summary of vaginal microbiota phyla (A) and the relative abundance of the top 50 most abundant genera (B) and species (C) (Average ± SD). (DOC) [file pone.0063514.s005.doc]

**Table S3:** Summary of vaginal microbiota phyla (A) and the relative abundance of the top 50 most abundant genera (B) and species (C) (Average ± SD).

**(A)**

| **Phylum** | **Premenopausal** | | | **Postmenopausal** | **MZ-discordant twins** | |
| --- | --- | --- | --- | --- | --- | --- |
|  | **HPV(-) (%)** | **HPV(+) (%)** | **CIN (%)** | **(%)** | **HPV(-) (%)** | **HPV(+) (%)** |
| Firmicutes | 77.4 (32.0) | 69.9 (33.4) | 85.7 (27.0) | 56.9 (35.2) | 90.5 (10.0) | 62.3 (36.0) |
| Bacteroidetes | 10.1 (19.5) | 10.9 (15.4) | 7.4 (15.9) | 10.6 (12.7) | 2.2 (3.9) | 13.2 (16.4) |
| Fusobacteria | 1.7 (3.9) | 8.9 (14.7) | 2.7 (5.6) | 0.1 (0.1) | 0.1 (0.2) | 13.4 (19.9) |
| Actinobacteria | 3.4 (6.7) | 6.3 (10.8) | 1.4 (2.0) | 6.3 (7.5) | 1.6 (3.1) | 8.8 (13.1) |
| Tenericutes | 3.7 (10.3) | 2.7 (6.3) | 1.4 (2.5) | 2.4 (5.1) | 4.7 (7.7) | 1.3 (2.6) |
| Proteobacteria | 1.2 (4.3) | 0.2 (0.3) | 0.3 (0.4) | 17.4 (23.9) | 0.5 (0.9) | 0.3 (0.4) |
| Other | 2.5 (5.7) | 1.1 (1.1) | 1.1 (1.5) | 6.3 (11.4) | 0.4 (0.4) | 0.9 (0.8) |

**(B)**

| **Genus** | **Premenopausal** | | | **Postmenopausal** | **MZ discordant twins** | |
| --- | --- | --- | --- | --- | --- | --- |
|  | **HPV(-)(%)** | **HPV(+)(%)** | **CIN (%)** | **(%)** | **HPV(-)(%)** | **HPV(+)(%)** |
| *Lactobacillus* | 63.6 (39.6) | 55.4 (41.0) | 57.6 (52.1) | 16.9 (36.7) | 76.8 (28.3) | 47.2 (46.9) |
| *Prevotella* | 8.6 (17.9) | 10.0 (14.5) | 6.9 (15.1) | 6.7 (8.8) | 1.0 (2.6) | 12.3 (15.5) |
| *Sneathia* | 1.7 (3.9) | 8.91 (4.7) | 2.7 (5.6) | 0.1 (0.1) | 0.1 (0.2) | 13.3 (19.9) |
| Clostridiales | 1.8 (7.4) | 3.71 (0.7) | 0.1 (0.3) | 0.5 (0.8) | 4.3 (12.6) | 6.3 15.0) |
| Lactobacillaceae | 0.1 (0.2) | < 0.1 (0.1) | < 0.1 (< 0.1) | 15.8 (33.7) | 0.1 (0.2) | < 0.1 (0.1) |
| Lachnospiraceae | 0.5 (1.9) | 1.5 (5.1) | 4.8 (10.7) | 1.9 (7.6) | < 0.1 (< 0.1) | 0.7 (1.9) |
| *Veillonella* | 2.2 (7.8) | 1.7 (6.9) | 9.5 (21.1) | 0.9 (3.1) | 2.6 (7.5) | 0.1 (0.1) |
| *Streptococcus* | 2.0 (8.1) | 0.1 (0.4) | 4.4 (9.9) | 4.6 (10.7) | 0.7 (2.1) | < 0.1 (< 0.1) |
| *Megasphaera* | 1.5 (4.8) | 2.1 (3.7) | 1.4 (3.1) | 0.4 (1.6) | 2.7 (7.9) | 3.8 (4.9) |
| *Atopobium* | 1.1 (2.9) | 3.4 (8.5) | 0.1 (0.2) | 1.0 (3.3) | 0.9 (2.6) | 4.9 (11.1) |
| *Ureaplasma* | 2.8 (9.8) | 1.3 (2.4) | 0.2 (0.2) | 2.2 (5.1) | 2.5 (5.3) | 1.2 (2.6) |
| *Dialister* | 0.9 (2.2) | 1.1 (1.5) | 3.3 (5.5) | 1.4 (1.9) | 0.1 (0.1) | 1.6 (1.7) |
| Lactobacillales | 2.2 (2.0) | 2.4 (2.5) | 1.5 (1.1) | 0.9 (1.2) | 1.1 (0.6) | 0.9 (0.9) |
| *Eggerthella* | 1.2 (3.4) | 2.3 (4.3) | 0.7 (1.5) | 0.3 (1.3) | < 0.1 (< 0.1) | 2.9 (5.0) |
| *Anaerococcus* | 0.6 (2.6) | 0.1 (0.1) | 0.7 (1.5) | 4.2 (6.6) | < 0.1 (0.1) | 0.1 (0.1) |
| Enterobacteriaceae | < 0.1 (0.2) | < 0.1 (< 0.1) | < 0.1 (< 0.1) | 5.01 (2.3) | < 0.1 (< 0.1) | < 0.1 (< 0.1) |
| Caulobacteraceae | 0.1 (0.1) | < 0.1 (0.1) | 0.1 (0.1) | 4.1 (11.6) | 0.1 (0.2) | < 0.1 (< 0.1) |
| Bacilli | 0.7 (0.7) | 0.7 (0.6) | 0.5 (0.5) | 0.3 (0.8) | 1.1 (0.8) | 0.3 (0.4) |
| *Porphyromonas* | 0.2(0.7) | 0.1 (0.3) | < 0.1 (< 0.1) | 1.7 (3.4) | < 0.1 (0.1) | 0.2 (0.4) |
| *Peptoniphilus* | 0.1 (0.6) | < 0.1 (< 0.1) | < 0.1 (< 0.1) | 2.4 (3.2) | < 0.1 (< 0.1) | < 0.1 (< 0.1) |
| Prevotellaceae | 0.3 (0.5) | 0.4 (0.6) | 0.2 (0.4) | 0.6 (0.9) | 0.1 (0.2) | 0.6 (0.8) |
| *Campylobacter* | 0.1 (0.2) | < 0.1 (0.2) | < 0.1 (< 0.1) | 2.6 (5.1) | < 0.1 (< 0.1) | 0.1 (0.3) |
| *Sphingomonas* | 0.1 (0.3) | < 0.1 (0.1) | 0.1 (0.1) | 2.3 (4.9) | < 0.1 (0.1) | < 0.1 (< 0.1) |
| *Mycoplasma* | 0.9 (4.0) | 1.4 (6.1) | 1.2 (2.6) | 0.1 (0.2) | 2.2 (6.5) | < 0.1 (< 0.1) |
| *Aerococcus* | 0.1 (0.5) | 0.1 (0.4) | 1.2 (2.7) | 0.4 (1.5) | 0.3 (0.9) | 0.3 (0.5) |
| *Gardnerella* | 0.5 (1.7) | 0.1 (0.3) | 0.3 (0.7) | 0.4 (1.1) | 0.6 (1.3) | 0.2 (0.4) |
| *Bacillus* | < 0.1 (< 0.1) | < 0.1 (< 0.1) | < 0.1 (< 0.1) | < 0.1 (< 0.1) | < 0.1 (< 0.1) | < 0.1 (< 0.1) |
| Sphingobacteriales | 0.4 (1.9) | < 0.1 (< 0.1) | < 0.1 (0.1) | 0.1 (0.1) | 1.1 (3.3) | < 0.1 (< 0.1) |
| *Bradyrhizobium* | 0.1 (0.4) | < 0.1 (0.1) | < 0.1 (0.1) | 1.1 (2.1) | 0.2 (0.6) | 0.1 (0.2) |
| Coriobacteriaceae | 0.2 (0.5) | 0.4 (0.6) | 0.2 (0.3) | 0.1 (0.2) | 0.1 (0.1) | 0.6 (0.8) |
| Veillonellaceae | 0.1 (0.2) | 0.2 (0.3) | 0.1 (0.3) | 0.2 (0.3) | 0.1 (0.3) | 0.3 (0.4) |
| *Finegoldia* | 0.1 (0.3) | < 0.1 (0.1) | < 0.1 (< 0.1) | 0.6 (1.4) | < 0.1 (0.1) | < 0.1 (< 0.1) |
| Ruminococcaceae | 0.2 (1.0) | 0.1 (0.2) | < 0.1 (< 0.1) | < 0.1 (0.1) | < 0.1 (0.1) | 0.2 (0.3) |
| *Parvimonas* | 0.1 (0.2) | 0.2 (0.4) | < 0.1 (0.1) | < 0.1 (0.1) | < 0.1 (0.1) | 0.2 (0.5) |
| *Agrococcus* | < 0.1 (< 0.1) | < 0.1 (< 0.1) | < 0.1 (< 0.1) | < 0.1 (< 0.1) | < 0.1 (< 0.1) | < 0.1 (< 0.1) |
| Alphaproteobacteria | < 0.1 (< 0.1 | < 0.1 (< 0.1) | 0.1 (0.2) | 0.2 (0.3) | < 0.1 (0.1) | < 0.1 (< 0.1) |
| Streptococcaceae | < 0.1 (< 0.1) | < 0.1 (< 0.1) | 0.1 (0.2) | 0.1 (0.4) | 0.1 (0.3) | < 0.1 (< 0.1) |
| *Propionibacterium* | < 0.1 (< 0.1) | < 0.1 (< 0.1) | < 0.1 (< 0.1) | 0.2 (0.5) | < 0.1 (< 0.1) | < 0.1 (< 0.1) |
| GpI | < 0.1 (< 0.1) | < 0.1 (< 0.1) | < 0.1 (< 0.1) | < 0.1 (< 0.1) | < 0.1 (< 0.1) | < 0.1 (< 0.1) |
| Bacillaceae | < 0.1 (< 0.1) | < 0.1 (< 0.1) | < 0.1 (< 0.1) | < 0.1 (< 0.1) | < 0.1 (< 0.1) | < 0.1 (< 0.1) |
| *Mobiluncus* | 0.3 (1.1) | 0.1 (0.3) | 0.1 (0.2) | 1.3 (3.9) | < 0.1 (< 0.1) | 0.1 (0.2) |
| *Staphylococcus* | < 0.1 (0.1) | < 0.1 (0.1) | < 0.1 (< 0.1) | 0.1 (0.2) | 0.1 (0.2) | 0.1 (0.2) |
| *Corynebacterium* | < 0.1 (0.1) | < 0.1 (< 0.1) | < 0.1 (< 0.1) | 1.6 (2.7) | < 0.1 (< 0.1) | < 0.1 (< 0.1) |
| Sphingomonadaceae | < 0.1 (< 0.1) | < 0.1 (< 0.1) | < 0.1 (< 0.1) | 0.8 (1.4) | < 0.1 (< 0.1) | < 0.1 (< 0.1) |
| *Moryella* | 0.1 (0.4) | < 0.1 (0.2) | < 0.1 (< 0.1) | 0.1 (0.2) | < 0.1 (< 0.1) | 0.1 (0.2) |
| *Shigella* | < 0.1 (0.1) | < 0.1 (< 0.1) | < 0.1 (< 0.1) | 0.5 (0.9) | < 0.1 (< 0.1) | < 0.1 (< 0.1) |
| Planctomycetaceae | < 0.1 (< 0.1) | < 0.1 (< 0.1) | < 0.1 (< 0.1) | < 0.1 (< 0.1) | < 0.1 (< 0.1) | < 0.1 (0.1) |
| *Gemella* | < 0.1 (< 0.1) | < 0.1 (0.1) | < 0.1 (< 0.1) | < 0.1 (< 0.1) | < 0.1 (< 0.1) | < 0.1 (< 0.1) |
| Porphyromonadaceae | < 0.1 (0.1) | < 0.1 (< 0.1) | < 0.1 (< 0.1) | 1.3 (2.6) | < 0.1 (< 0.1) | < 0.1 (< 0.1) |
| Bacteria, other | 4.4 (5.7) | 2.0 (0.9) | 1.7 (1.5) | 13.9 (7.3) | 0.7 (0.4) | 1.3 (0.7) |

**(C)**

| **Species** | **Premenopausal** | | | **Postmenopausal** | **MZ discordant twins** | |
| --- | --- | --- | --- | --- | --- | --- |
|  | **HPV(-)(%)** | **HPV(+)(%)** | **CIN (%)** | **(%)** | **HPV(-)(%)** | **HPV(+)(%)** |
| *Lactobacillus iners* | 32.1 (35.8) | 36.6 (38.2) | 42.0 (46.3) | 16.6 (34.3) | 41.6 (30.6) | 10.5 (16.0) |
| *Lactobacillus crispatus* | 24.9 (36.3) | 21.5 (33.9) | 17.2 (33.7) | 16.2 (35.8) | 26.7 (33.9) | 32.4 (37.3) |
| *Atopobium vaginae* | 1.3 (3.4) | 3.8 (11.5) | 0.1 (0.2) | 1.0 (3.4) | 0.9 (2.6) | 7.4 (16.7) |
| *Ureaplasma parvum* | 2.7 (9.6) | 1.0 (2.0) | 0.2 (0.2) | 2.1 (5.0) | 2.5 (5.0) | 1.3 (2.5) |
| *Lactobacillus jensenii* | 2.4 (7.9) | 1.3 (4.8) | 0.1 (0.1) | 0.2 (1.0) | 2.6 (6.8) | < 0.1 (0.1) |
| *Leptotrichia amnionii* | 0.4 (1.4) | 3.2 (6.3) | 3.0 (5.9) | < 0.1 (< 0.1) | < 0.1 (0.2) | 5.0 (8.3) |
| *Mycoplasma hominis* | 0.9 (3.8) | 1.5 (5.7) | 1.5 (3.1) | < 0.1 (<0.1) | 2.3 (6.4) | < 0.1 (< 0.1) |
| *Bacteroides ureolyticus* | < 0.1 (0.2) | < 0.1 (< 0.1) | 0.0 (0.0) | 2.4 (5.1) | < 0.1 (< 0.1) | 0.1 (0.3) |
| *Sphingomonas echinoides* | < 0.1 (0.1) | < 0.1 (< 0.1) | < 0.1 (0.1) | 2.4 (5.3) | < 0.1 (< 0.1) | < 0.1 (< 0.1) |
| *Peptoniphilus asaccharolyticus* | 0.1 (0.5) | < 0.1 (< 0.1) | < 0.1 (< 0.1) | 2.2 (2.9) | < 0.1 (< 0.1) | < 0.1 (< 0.1) |
| *Streptococcus agalactiae* | 0.9 (4.3) | < 0.1 (< 0.1) | 4.6 (9.1) | 0.9 (3.4) | < 0.1 (< 0.1) | < 0.1 (< 0.1) |
| *Streptococcus pseudopneumoniae* | < 0.1 (< 0.1) | < 0.1 (0.3) | < 0.1 (< 0.1) | 2.0 (5.9) | < 0.1 (< 0.1) | < 0.1 (< 0.1) |
| *Streptococcus anginosus* | 0.1 (0.5) | < 0.1 (< 0.1) | < 0.1 (< 0.1) | 1.8 (6.0) | < 0.1 (< 0.1) | < 0.1 (< 0.1) |
| *Gardnerella vaginalis* | 0.6 (1.6) | 0.1 (0.27) | 0.3 (0.6) | 0.4 (1.0) | 0.6 (1.2) | 0.2 (0.4) |
| *Mobiluncus mulieris* | 0.2 (1.1) | < 0.1 (0.2) | 0.1 (0.2) | 0.9 (3.8) | 0.0 (0.0) | < 0.1 (< 0.1) |
| *Afipiagenosp.1* | < 0.1 (0.3) | < 0.1 (< 0.1) | < 0.1 (< 0.1) | 1.1 (2.1) | 0.2 (0.5) | < 0.1 (0.1) |
| *Dialistermicr aerophilus* | 0.6 (2.4) | 0.2 (0.2) | 2.7 (5.1) | 0.1 (0.2) | < 0.1 (< 0.1) | 0.3 (0.3) |
| *Anaerococcus hydrogenalis* | < 0.1 (0.1) | < 0.1 (< 0.1) | < 0.1 (< 0.1) | 1.0 (1.6) | < 0.1 (< 0.1) | 0.0 (0.0) |
| *Caulobacter leidyia* | < 0.1 (< 0.1) | < 0.1 (< 0.1) | 0.1 (0.2) | 0.8 (1.4) | < 0.1 (< 0.1) | 0.0 (0.0) |
| *Mobiluncus curtisii* | 0.1 (0.6) | < 0.1 (0.3) | < 0.1 (< 0.1) | 0.4 (0.9) | 0.0 (0.0) | < 0.1 (0.2) |
| *Anaerococcus prevotii* | 0.1 (0.4) | < 0.1 (< 0.1) | < 0.1 (< 0.1) | 0.4 (0.7) | 0.0 (0.0) | < 0.1 (< 0.1) |
| *Actinobaculum schaalii* | 0.0 (0.0) | < 0.1 (< 0.1) | < 0.1 (< 0.1) | 0.3 (0.9) | 0.0 (0.0) | 0.0 (0.0) |
| *Propionibacterium acnes* | < 0.1 (< 0.1) | < 0.1 (< 0.1) | < 0.1 (< 0.1) | 0.2 (0.5) | < 0.1 (< 0.1) | < 0.1 (< 0.1) |
| *Corynebacterium acnes* | < 0.1 (< 0.2) | < 0.1 (< 0.1) | < 0.1 (< 0.1) | 0.2 (0.8) | < 0.1 (< 0.1) | < 0.1 (< 0.1) |
